# Identification of Reticulocyte Binding Domain of *Plasmodium ovale curtisi* Duffy Binding Protein (PocDBP) Involved in Reticulocyte Invasion

**DOI:** 10.3389/fcimb.2021.764293

**Published:** 2021-12-10

**Authors:** Mohammad Rafiul Hoque, Myat Htut Nyunt, Jin-Hee Han, Fauzi Muh, Seong-Kyun Lee, Ji-Hoon Park, Feng Lu, Won Sun Park, Eun-Taek Han, Sunghun Na

**Affiliations:** ^1^ Department of Medical Environmental Biology and Tropical Medicine, Kangwon National University School of Medicine, Chuncheon, South Korea; ^2^ Department of Medical Research, Yangon, Myanmar; ^3^ School of Medicine, Yangzhou University, Jiangsu Key Laboratory of Experimental & Translational Non-coding RNA Research, Yangzhou, China; ^4^ Department of Physiology, School of Medicine, Kangwon National University, Chuncheon, South Korea; ^5^ Department of Obstetrics and Gynecology, Kangwon National University School of Medicine, Chuncheon, South Korea

**Keywords:** *P. ovale curtisi*, malaria, invasion, PocDBP-RII, reticulocyte binding

## Abstract

The *Plasmodium ovale curtisi* (Poc) prevalence has increased substantially in sub-Saharan African countries as well as regions of Southeast Asia. Poc parasite biology has not been explored much to date; in particular, the invasion mechanism of this malaria parasite remains unclear. In this study, the binding domain of the Duffy binding protein of *P. ovale curtisi* (PocDBP) was characterized as an important ligand for reticulocyte invasion. The homologous region of the *P. vivax* Duffy binding protein in PocDBP, named PocDBP-RII herein, was selected, and the recombinant PocDBP-RII protein was expressed in an *Escherichia coli* system. This was used to analyze reticulocyte binding activity using fluorescence-activated cell sorting and immune serum production in rabbits. The binding specificity was proven by treating reticulocytes with trypsin, chymotrypsin and neuraminidase. The amino acid sequence homology in the N-terminal Cys-rich region was found to be ~ 44% between PvDBP and PocDBP. The reticulocyte binding activity of PocDBP-RII was significantly higher than the erythrocyte binding activity and was concentration dependent. Erythrocyte binding was reduced significantly by chymotrypsin treatment and inhibited by an anti-PocDBP-RII antibody. This finding suggests that PocDBP may be an important ligand in the reticulocyte invasion process of *P. ovale curtisi.*

## Introduction

Malaria is a leading global public health concern, especially in Africa (World Health Organization, 2020). The World Health Organization reported that approximately 229 million cases of malaria occurred worldwide in 2019, compared with 218 million cases in 2018, and estimated that 409,000 deaths occurred from malaria in 2019, compared to 411,000 in 2018 (World Health Organization, 2020). Among the five *Plasmodium* spp. parasites, *Plasmodium falciparum* is the deadliest parasite that accounts for substantial death each year, primarily in Africa, while *Plasmodium vivax* causes a benign form of the disease and is widely distributed in Southeast Asia and areas of the Amazon Basin of South America (Gething et al., 2011; Howes et al., 2016). Compared to these two species, *Plasmodium ovale* has become much less prevalent, presumably, in recent decades (Hawadak et al., 2021; Mahittikorn et al., 2021). However, the number of ovale malaria cases has increased in African countries and in Chinese individuals returning from Africa (Roucher et al., 2014; Cao et al., 2016). Although the primary emphasis for malaria research has been on falciparum malaria and noticeably on vivax malaria, *P. ovale* malaria is typically neglected.

Due to its low parasite density, milder clinical manifestations, and morphological resemblance to *P. vivax* in microscopy examination, *P. ovale* tends to be mis- or underdiagnosed (Phuong et al., 2016; Yerlikaya et al., 2018). However, recent findings indicated that *P. ovale* could be divided into two genetically distinct sympatric subspecies named *P. ovale curtisi* and *P. ovale wallikeri* (Sutherland et al., 2010; Oguike et al., 2011; Fuehrer and Noedl, 2014). A growing body of evidence has indicated a significant increase in *P. ovale curtisi* and *P. ovale wallikeri* cases worldwide, especially in African countries (Mahittikorn et al., 2021). Because of the low endemicity and lack of an *in vitro* cultivation system, the biology of the *P. ovale* subspecies has not been well investigated to date. Moreover, the genome sequences of these two parasites were published recently, allowing in-depth exploration of parasite pathophysiology, particularly the blood-stage invasion process (Ansari et al., 2016; Rutledge et al., 2017).

Several blood-stage ligands of *Plasmodium* spp. are responsible for invasion into erythrocytes (Weiss et al., 2015). *P. vivax* and *P. knowlesi* depend primarily on the interactions of Duffy binding protein (DBP) and Duffy antigen receptor for chemokine (DARC) for reticulocyte invasion (Chitnis et al., 1996; Kanjee et al., 2021). A previous study reported that *P. ovale* invades reticulocytes (Collins and Jeffery, 2005). However, there is a lack of *in vitro* experimental evidence regarding the *P. ovale curtisi* invasion pathway. Here, we performed functional characterization of one of the invasion ligands, *P. ovale curtisi* Duffy binding protein domain region II (PocDBP-RII), which is probably responsible for host red blood cell invasion.

Merozoite invasion is a multistep sequential process of molecular interactions between merozoite ligands and host receptors present on the erythrocyte membrane (Cowman and Crabb, 2006; Weiss et al., 2015; Collins et al., 2020). The invasion process is broadly categorized into three phases: initial attachment, invasion, and echinocytosis (Cowman et al., 2017). Initial attachment to erythrocytes mediated by merozoite surface proteins is usually initiated by merozoite surface protein-1 (MSP-1) and can occur at any point in erythrocytes (Cowman et al., 2017). Reorientation facilitates further close interaction between the apical end of the merozoite and the erythrocyte surface, followed by robust deformation at a place of contact. A tight junction is formed by apical membrane antigen-1 (AMA-1) and rhoptry neck protein 2 (RON2), and the invasion process is propelled by an actin-myosin motor (Srinivasan et al., 2011). This step leads to shedding of the fuzzy coating of the merozoite surface by proteases. Finally, at the echinocytosis phase, the parasite seals itself from the host cell cytoplasm, forms a parasitophorous vacuole, and stops the invasion process (Srinivasan et al., 2011).

Several proteins secreted from apical secretory organelles play a key role in successful invasion (Cowman et al., 2017). However, the detailed mechanism of this invasion process is not yet fully understood (Cowman and Crabb, 2006; Cowman et al., 2017). Moreover, primary invasion ligands vary among *Plasmodium* spp. (Cowman et al., 2017). According to a previous report, *P. ovale curtisi* has unique characteristics and a reticulocyte preference for invasion of erythrocytes (Collins and Jeffery, 2005). The lack of a continuous culture system for *P. ovale* has hindered the investigation of the exact mechanism of invasion (Schuster, 2002). Functional characterization of individual proteins might be an indirect method to overcome this technical difficulty. Thus, in the current study, we aimed to determine the functional activity of PocDBP-RII. Our results showed that PocDBP-RII has a preference for reticulocyte binding.

## Materials and Methods

### Expression and Purification of the Recombinant PocDBP-RII and PvDBP-RII Protein

The gene encoding region II (RII) of PocDBP (aa 182 to 506) was amplified using nested PCR. Genomic DNA was extracted from whole-blood samples from a parasite-infected patient, which was kindly provided by Jiangsu Institute of Parasitic Diseases, Wuxi, China. Primer sets for nested PCR were designed based on the *pocdbp* (*PocGH01_00129200)* sequence. The primers used were as follows: Nest 1 F: *tcgcggatccgaattc* GCTTTTAGAGATGTTCCTAATTATGG; Nest 1 R: *ggtggtggtgctcgag* TTTTATTCCTTTCTGCGCG; Nest 2 F: *tcgcggatccgaattc* AATATTACAAACAATGATGTAAATTATGT; Nest 2 R: *ggtggtggtgctcgag* TTTTATTCCTTTCTGCGCG. The restriction enzymes *EcoR*I and *Xho*I are indicated as italicized and underlined letters. PCR amplification was performed using high-fidelity Phusion DNA polymerase (New England Biolabs Inc., Ipswich, MA). Each reaction consisted of a total volume of 20 µl containing 7.5 mM MgCl_2_, 2.5 mM dNTPs, 0.5 µl of sense and antisense primers (100 pmol/µl), 0.2 µl of high-fidelity Phusion DNA polymerase (2 U/µl) and 2 µl of gDNA as template. The PCR thermal cycling conditions were set as follows: initial denaturation at 95°C for 5 min, followed by 35 cycles of 95°C for 30 s, 59°C for 30 s and 72°C for 1 min and a final extension at 72°C for 10 min. Amplicons were gel purified using a DNA purification kit (Macherey–Nagel, Duren, Germany) according to the manufacturer’s instructions and ligated into the pET28a (+) expression vector (Novagen, Madison, WI) with a C-terminal His-tag. The obtained purified plasmid DNA sequence was confirmed by sequencing analysis and transformed into BL21 (DE3) competent cells (Novagen). Isopropyl-β-d-thiogalactopyranoside (IPTG; 1.0 mM; Sigma-Aldrich Co., St. Louis, MO) was used to induce recombinant protein expression. Protein solubilization, purification, and refolding were performed as previously described (Singh et al., 2001). The refolded proteins were eluted by ion-exchange chromatography (HiTrap™ SP FF; GE Healthcare Life Sciences, Chicago, IL) using 1 M NaCl. GST-His (glutathione S-transferase 6 X His tag) protein was expressed as per manufacturer’s protocol (GST Gene Fusion system, GE Healthcare Life Sciences, Uppsala, Sweden). Recombinant PvDBP-RII protein was expressed and purified as described elsewhere (Singh et al., 2001).

### SDS-PAGE and Western Blot Analyses

The refolded recombinant PocDBP-RII protein was separated by 8% SDS-PAGE and stained with 0.25% Coomassie brilliant blue (Sigma-Aldrich Co.). Briefly, 5 μg of refolded protein was incubated with 10 mM dithiothreitol (DTT, reducing condition) at 37°C for 1 hr, followed by the addition of 2× loading dye containing the reducing agent 2-mercaptoethanol; in addition, 5 μg of protein was processed without DTT (nonreducing condition) and with the 2× loading dye without reducing agent. The samples were heated at 100°C for 4 min. For the Western blot analysis, the proteins were electrotransferred to 0.45 μm PVDF membranes (Millipore, Bedford, MA) by electrophoresis in semidry transfer buffer (50 mM Tris, 190 mM glycine, 3.5 mM SDS, 20% methanol) with a continual current of 370 mA for 40 min using a semidry transfer system (ATTO Corp., Tokyo, Japan). Then, the membrane was incubated with blocking buffer (5% skim milk in PBS containing 0.2% Tween 20) and then incubated with a primary anti-penta-histidine antibody (1:2000) and rabbit immune serum (1:1000), followed by incubation with a secondary IRDye^®^ goat anti-rabbit antibody (1:10,000 dilution) (LI-COR^®^ Bioscience, Lincoln, NE). Data analysis was performed using an Odyssey infrared imaging system and the company-recommended software (LI-COR^®^ Bioscience).

### Animal Immune Sera Production

One Japanese white rabbit was used to produce an anti-PocDBP-RII antibody. Two hundred and fifty micrograms of purified protein with complete Freund’s adjuvant (Sigma-Aldrich Co.) was injected subcutaneously, followed by treatment with 250 µg of incomplete Freund’s adjuvant for subsequent boosting. All immunizations were administered 3 times at 3-week intervals. Antisera were collected two weeks after the final boost. Anti-PvDBP-RII antibody generation was performed as described in our previous study (Han et al., 2016). Total IgG was purified from 1 mL of anti-PvDBP-RII and anti-PocDBP-RII rabbit immune serum by using a protein G HP column as per manufacturer’s protocol (GE Healthcare Life Sciences) as described elsewhere (Muh et al., 2018). All the experimental protocols were approved by the Kangwon National University Animal Care and Use Committee, and the experiments were conducted according to the Ethical Guidelines for Animal Experiments of Kangwon National University (KIACUC-16-0157).

### Cord Blood Samples

Umbilical cord blood samples were collected in a 10 ml heparin tube (BD Vacutainer^®^, Becton-Dickinson Co., Franklin Lakes, NJ). The relevant guidelines and regulations were followed to conduct all the experiments, and the human sample-related experimental protocols were approved by the Kangwon National University Hospital Ethical Committee (IRB No. 2014-08-008-006). Written informed consent was obtained from all the subjects.

### Reticulocyte Enrichment From Cord Blood

Reticulocytes were enriched from umbilical cord blood using a cushion of 19% Nycodenz solution (Axis-Shield, Oslo, Norway) in high-KCl buffer *via* gradient centrifugation. Upon receipt, the fresh cord blood was washed twice with incomplete RPMI 1640 medium, and white blood cells (WBCs) were removed using an NWF filter (Zhixing Bio Co., Ltd., Bengbu, China). WBC-free packed cells were then resuspended in high-KCl buffer (115 mM KCl) (pH 7.4), followed by incubation at 4°C for 3 hr with rotation. Three milliliters of prewarmed Nicodenz solution (19%) was transferred into 15 ml tubes. Then, 5 ml of the RBC-high-KCl buffer mixture was poured on top of the Nicodenz cushion and centrifuged for 30 min at 3000 ×g without braking. The reticulocytes were harvested from the interface layer between Nicodenz and high-KCl buffer and washed three times with incomplete RPMI 1640 medium. Reticulocyte purity was determined from thin blood smears with new methylene blue staining by light microscopy and thiazole orange (TO) staining of the harvested reticulocytes. A total of 100,000 events were obtained per sample using a FACS Accuri™ C6 Flow Cytometer (Becton-Dickinson Co., Mansfield, NJ).

### Enzyme Treatment of RBCs

Enriched reticulocytes were prepared with up to 50% hematocrit. Then, the erythrocytes were washed with 500 μl of incomplete RPMI 1640 medium twice by centrifugation at 500 ×g for 3 min at 4°C. Then, the erythrocytes were treated with either neuraminidase (100 mU; from *Vibrio cholerae*, Sigma-Aldrich Co.), trypsin (0.5 mg; from bovine pancreas, Sigma-Aldrich Co.) or chymotrypsin (0.5 mg; from bovine pancreas, Sigma-Aldrich Co.) at 37°C on a rotator for 1 hr. After enzyme treatment, chymotrypsin- and trypsin-treated RBCs were incubated with a trypsin inhibitor from soybean (*Glycine max*) (Sigma-Aldrich Co.) at 37°C for 10 min and subsequently washed three times with 10 ml of incomplete RPMI 1640 medium. Packed cells were prepared at a concentration of 1 × 10^6^ cell/ml and used for flow cytometry analysis.

### Reticulocyte Binding Assay by Flow Cytometry

The erythrocyte binding assay was performed as described previously (Tran et al., 2005). Briefly, a gradient concentration of purified PocDBP-RII protein was incubated with 1 × 10^6^/ml cells or the same concentration of reticulocytes treated with each enzyme for 3 hr at 25°C. The PvDBP-RII and GST-His protein reticulocyte binding activities were used as the experimental controls. The samples were washed with 200 µl of PBS (1% BSA) two times, followed by incubation with diluted (1:50) mouse anti-penta-His Alexa Fluor 647-conjugated monoclonal antibody (Qiagen, Hilden, Germany) for 1 hr at 4°C in the dark. The samples were washed three times with PBS (1% BSA) and incubated with TO (Becton-Dickinson Co., San Jose, CA) for 30 min at 25°C. A total of 100,000 events were counted per sample using a FACS Accuri™ C6 Flow Cytometer (Becton-Dickinson Co.). FlowJo 7.6 (Treestar, Ashland, OR) was used to analyze the flow cytometric results. Unstained cells and cells singly stained with TO represented normocytes and reticulocytes, respectively.

### Three-Dimensional Structure Prediction

Three-dimensional (3-D) structure modeling and validation of PocDBP-RII and PvDBP-RII were performed by homology-based modeler software. Satisfactory structural templates were explored and modeled using SWISS-MODEL (Biasini et al., 2014). The error residues were refined by using Galaxy Refine (Ko et al., 2012). Finally, all the structures were visualized by UCSF CHIMERA (Huang et al., 2014).

### Statistical Analysis

Data analysis was performed using GraphPad Prism (GraphPad Software, San Diego, CA). Student’s *t*-test was used to compare the experimentally measured values of different groups. Values of *p* < 0.05 indicated significant differences.

## Results

### Schematic Structure of PocDBP

**Figure 1:**
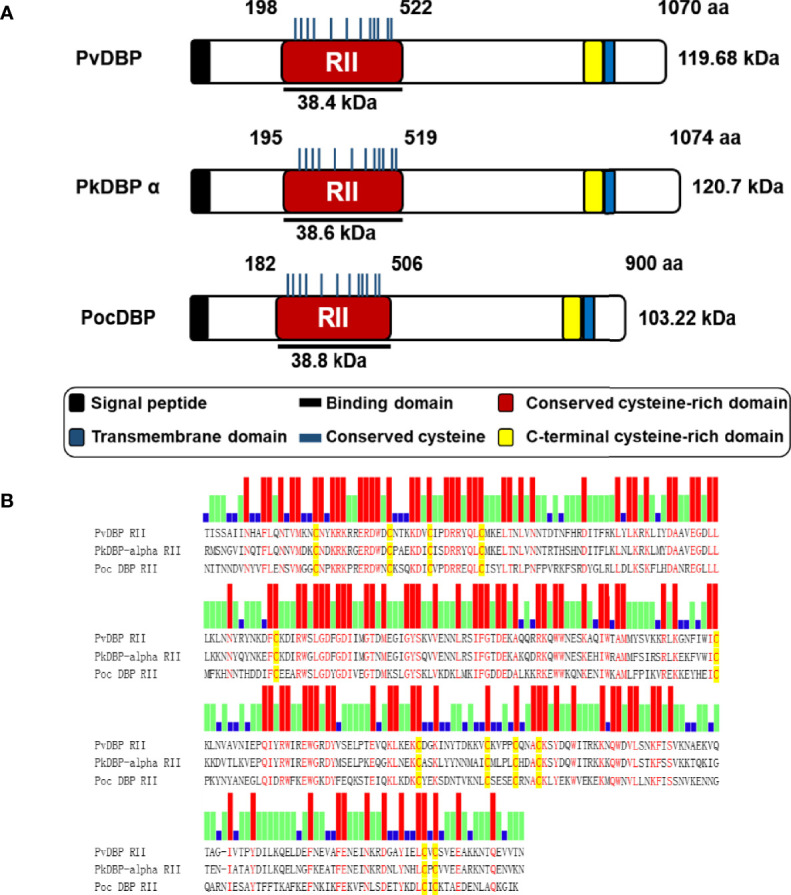
Schematic depiction and sequence alignments of DBP proteins. **(A)** Schematic depiction of PvDBP, PkDBP-α and PocDBP. The PocDBP-RII (aa 182–506) domain was expressed using bacterial expression systems. The signal peptide (black box), transmembrane domain (blue box), recombinant protein expression (black bar) and functional erythrocyte-binding domain (red bar) are indicated. **(B)** Clustal alignment of the PvDBP-RII, PkDBP-α-RII, and PocDBP-RII homologous sequences. The red bar represents the conserved identical amino acids in the alignment of the three proteins, the green bar represents identical amino acids in two proteins, and the blue bar represents diverse amino acids in the three proteins. The yellow shading denotes conserved cysteine residues.

The *pocdbp* gene sequence encodes a moderate-sized protein (900 amino acids) with a predicted molecular weight of 103.22 kDa (**Figure 1A**). The gene consists of 5 exons encoding a signal sequence, a transmembrane domain and 22 cysteine residues. The putative functional domain site of PocDBP-RII is defined based on the PvDBP-RII homolog site as an erythrocyte-binding domain. Twelve cysteine residues are conserved in the RII domain of the *pocdbp* gene, similar to PvDBP-RII and PkDBP-α RII, which is probably indicative of similar functions. The sequence alignments of PvDBP-RII, PkDBP-α-RII, and PocDBP-RII were generated using Clustal W, revealing that PocDBP-RII shares 44.4% and 40.4% amino acid sequence identity with PvDBP-RII and PkDBP-α-RII, respectively (**Figure 1B**).

### Three-Dimensional Structure Analysis

**Figure 2:**
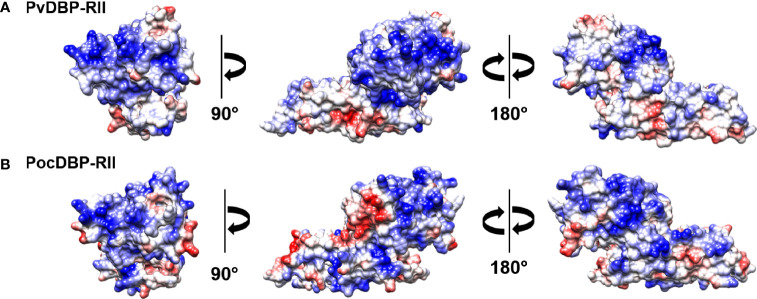
Three-dimensional structure of DBP-RII. The electrostatic surfaces of **(A)** PvDBP-RII and **(B)** PocDBP-RII with positive (blue) and negative (red) charges are shown.

The three-dimensional structure analysis clearly indicated the similar shape of the binding pocket and structure between PvDBP-RII and PocDBP-RII (**Figure 2**). Based on the electric charge, the structure is represented mainly by two distinct parts (**Figure 2**). Positively charged PvDBP-RII structures are well known for binding to reticulocytes (Batchelor et al., 2011). Despite low sequence identity, the PocDBP-RII structure was very similar to that of PvDBP-RII (**Figure 2**), which might indicate similar functional activities.

### Expression and Purification of the Recombinant PocDBP-RII and PvDBP-RII Protein

**Figure 3:**
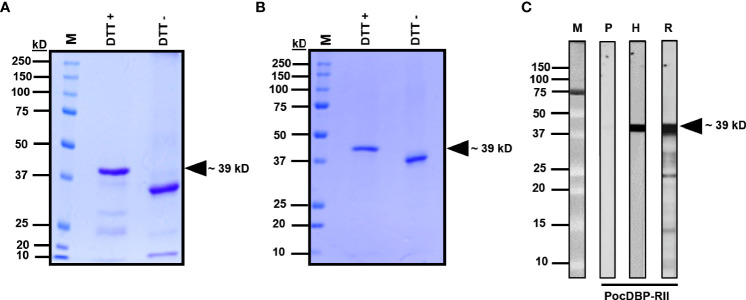
Recombinant PvDBP-RII and PocDBP-RII protein expression and evaluation of refolding. **(A, B)** Coomassie blue-stained SDS-PAGE gel of recombinant PvDBP-RII and PocDBP-RII. Dithiothreitol (DTT) (-) and DTT (+) indicate the refolded protein and denatured protein, respectively, before and after treatment with 10 mM DTT. M, protein size markers. **(C)** Western blot analysis of the recombinant PocDBP-RII protein probed with antibodies. Black-head arrows indicate specific target bands. H, anti-His antibody; R, αPocDBPRII IgG; P, preimmune rabbit IgG.

The recombinant PvDBP-RII and PocDBP-RII protein was purified from inclusion bodies after bacterial expression and refolded by rapid dilution as previously published (Singh et al., 2001). The recombinant PocDBP-RII protein was used for rabbit immunization. Evidence for refolding of the purified recombinant PvDBP-RII and PocDBP-RII protein was shown by SDS-PAGE analysis (**Figures 3A, B**) (Muh et al., 2018). Different mobilities of the refolded protein between reducing (DTT +) and nonreducing conditions (DTT −) indicated that the native protein had been formed correctly after refolding (**Figures 3A, B**). Anti-PocDBP-RII IgG and anti-His antibodies could be used to detect the recombinant proteins by Western blot analysis (**Figure 3C**). These results suggest that immune serum raised against PocDBP-RII can recognize the recombinant protein.

### Reticulocyte-Binding Activity of PocDBP-RII

**Figure 4:**
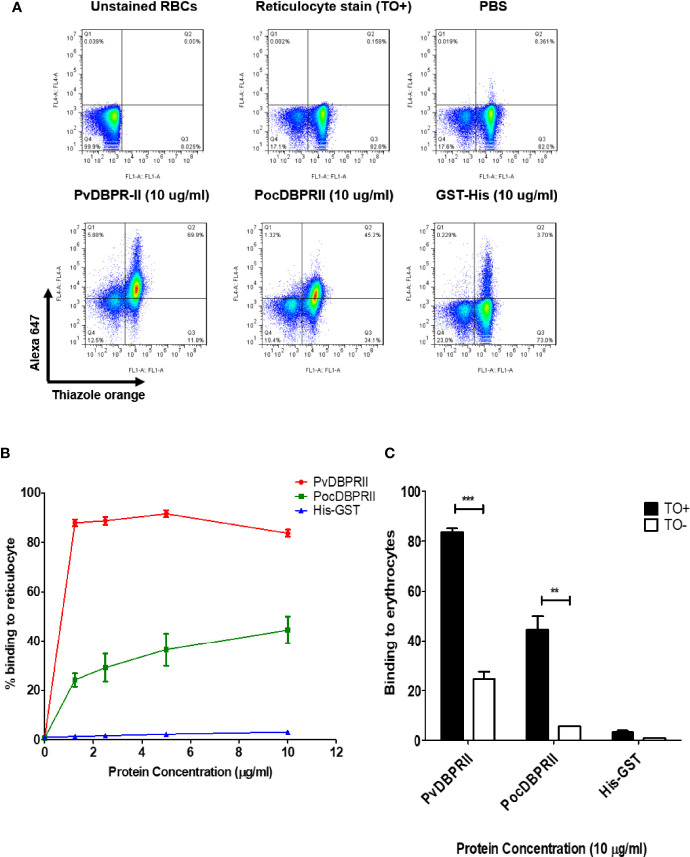
Reticulocyte-binding activity of recombinant PocDBP-RII in the FACS-based assay. **(A)** Dot plot patterns. Unstained RBCs, gating control (upper left); enriched reticulocytes, thiazole orange, TO+ (upper center); binding control without protein, phosphate-buffered saline (PBS) in the added fractions (upper right). Recombinant proteins (10 μg/ml) added to the test samples are shown in the lower image. **(B)** Reticulocyte-binding assay showing the total binding percentage of reticulocytes with gradient concentrations of the proteins. The PvDBP-RII and GST-His proteins were used as positive and negative controls, respectively. The data are shown as the mean ± standard deviation (SD) of at least three independent experiments. **(C)** The bar chart shows the binding of the PvDBP-RII, PocDBPRII and GST-His proteins (each at 10 μg/ml) to TO (+) (normocytes) and TO (-) (reticulocytes). Significant differences are shown as double asterisks, p < 0.002; triple asterisks <0.0001. The data are shown as the mean ± SD of at least three independent experiments.

A fluorescence-activated cell sorting (FACS)-based binding assay was used for reticulocyte-binding activity evaluation. The PvDBP-RII and His-tagged GST proteins were used as positive and negative controls, respectively, for validation of binding activity. Reticulocytes (61.93% on average) were enriched from cord blood and used for the binding assay (**Figure 4A**). PvDBP-RII bound strongly to reticulocytes at a protein concentration of 1.25 μg/ml, and binding saturation was observed at a concentration of approximately 5 μg/ml (**Figure 4B**). The binding of PocDBP-RII to reticulocytes was shown to increase in a concentration-dependent manner and was saturated at a concentration of 10 μg/ml (**Figure 4B**). PvDBP-RII binding was detected at a concentration of 10 μg/ml, with a mean binding activity of 83.74% ± 1.24% with reticulocytes and 24.59% ± 2.90% with normocytes (*p* < 0.0001), which represented a 3.4-fold increase in reticulocytes (**Figure 4C**). The PocDBP-RII protein was bound explicitly to reticulocytes at a concentration of 10 μg/ml with a mean binding activity of 44.55% ± 5.44%, which was a 8.0 fold higher than the normocyte binding activity (*p* < 0.002) (**Figure 4C**). The GST-His protein normocyte binding activity was similar to that of reticulocytes and was below the cutoff percentage, which indicated that there was no binding activity with normocytes (**Figure 4C**). The GST-His protein binding activity was measured as a negative control.

**Figure 5:**
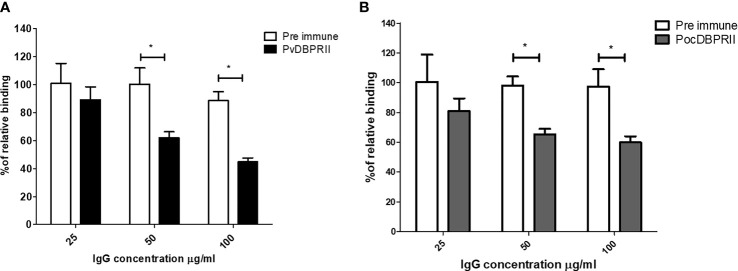
Binding inhibition assay with the anti-PocDBP-RII antibody. Rabbit anti-PvDBP-RII **(A)** and anti-PocDBP-RII **(B)** antibodies were tested against homologous proteins in a flow cytometry binding assay for inhibition of PvDBP-RII and PocDBP-RII reticulocyte binding. Each bar represents the percent binding to reticulocytes in the presence of immune IgG relative to preimmune IgG. Significant differences are shown as single asterisks, p < 0.05. The data are shown as the mean ± standard deviation of at least two independent experiments.

**Figure 6:**
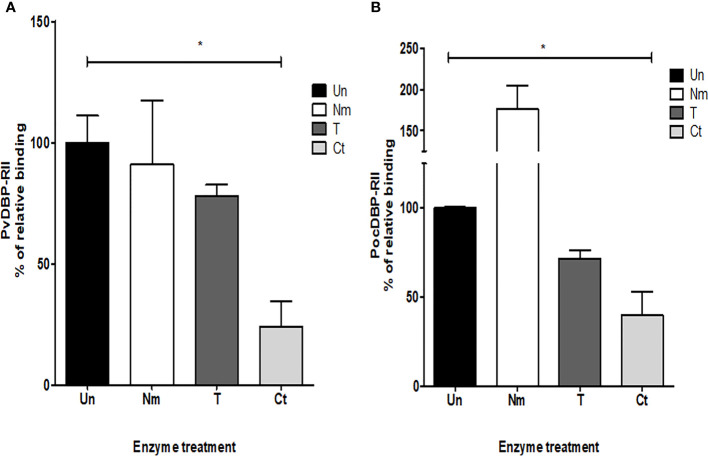
Enzyme-treated reticulocyte binding assay. A flow cytometry-based erythrocyte binding assay of PvDBP-RII **(A)** and PocDBP-RII **(B)** was performed with different enzyme-treated erythrocytes: Un, untreated RBCs; Nm, neuraminidase; T, trypsin; Ct, chymotrypsin. Significant differences are shown as single asterisks, *p* < 0.05. The data are shown as the mean ± standard deviation of at least two independent experiments.

The reticulocyte binding specificity was confirmed by an antibody inhibition assay. The anti-PocDBP-RII IgG antibody was able to inhibit reticulocyte binding in a concentration-dependent manner (**Figure 5**). Binding inhibition was significantly different at concentration of 50 μg/ml and 100 μg/ml of anti-PocDBP-RII IgG (*p* = 0.0228 and *p* = 0.0496 respectively) as compared to preimmune rabbit IgG. Reticulocytes treated with trypsin, chymotrypsin, and neuraminidase were also used to check the binding specificity of the recombinant PocDBP-RII protein. The PvDBP-RII reticulocyte binding activity with chymotrypsin-treated reticulocytes was inhibited by three-fourths compared to that of untreated PvDBP-RII (percentage of relative binding, mean ± SD: 23.87% ± 7.49%); this binding activity was significantly different (*p* = 0.0196) from the binding activity for normal reticulocytes. A similar binding pattern was also observed for the PocDBP-RII protein. The PocDBP-RII binding activity (39.8% ± 9.19%) was significantly inhibited by chymotrypsin (*p* = 0.0226) (**Figure 6**).

## Discussion

Merozoite invasion into erythrocytes in blood-stage parasites is critical for malaria infection (Cowman et al., 2017). Notable progress has already made in understanding this mechanism in the last few decades, especially for *P. falciparum* and *P. vivax* (Wright and Rayner, 2014; Cowman et al., 2017; Scully et al., 2017). However, only a few invasion ligands along with their corresponding receptors have been identified as critically important for the erythrocyte invasion process (Dolan et al., 1994; Chitnis et al., 1996; Tham et al., 2010; Crosnier et al., 2011; Baldwin et al., 2015; Gruszczyk et al., 2018). Compared to *P. falciparum* and *P. vivax*, the invasion ligands for *P. ovale curtisi* have not yet been studied. In this study, we characterized PocDBP-RII as one of the domains essential for the invasion process.

This study demonstrates that the PocDBP-RII domain preferentially binds to reticulocytes rather than erythrocytes in a concentration-dependent manner. The *P. vivax* and *P. knowlesi* parasites use DBP and DBP-α, respectively, for erythrocyte invasion. PocDBP is a homolog of the DBL family of the *P. vivax* and *P. knowlesi* DBPs. A similar binding preference of PocDBP to reticulocytes was identified in the current study, which supports the previous hypothesis that *P. ovale* uses reticulocytes for invasion (Collins and Jeffery, 2005). However, the highest binding strength of PocDBP was found at 10 µg/ml (**Figure 3C**), which was approximately half the binding strength of PvDBP-RII. We speculated that difference in binding frequency to reticulocyte between PvDBP-RII and PocDBP-RII may suggest different receptor-ligand interaction (França et al., 2016; Han et al., 2016; Gruszczyk et al., 2018). Likewise, Poc parasites may use PocDBP-RII as a secondary pathway for invasion whereas identification of primary invasion ligands exploited by Poc parasites require further exploration. A similar binding activity was previously identified for the PvRBP1b-32 protein (Han et al., 2016). Moreover, compared to *P. vivax*, *P. ovale curtisi* has an additional DBL protein, PocDBP2, which is homologous to the *P. vivax* erythrocyte binding protein (PvEBP) (Ansari et al., 2016; Rutledge et al., 2017). PvEBP also prefers to bind to reticulocytes rather than normocytes, although the binding activity observed was relatively low (Ntumngia et al., 2016). In the current study, the PocDBP-RII domain function was characterized, but the function of another DBL family member, PocDBP2, has yet to be studied to determine the complete role of the DBL family in the case of *P. ovale curtisi*.

Although similar to *P. vivax* DBP, PocDBP-RII shows a preference for reticulocytes over normocytes, and it was reported that *P. ovale* spp. infection was not restricted by DARC on the erythrocyte surface for complete invasion (Jeffery et al., 1954; Jeffery et al., 1955). A previous study hypothesized that the two sympatric *P. ovale* spp. might use the receptor-ligand mechanism to invade reticulocytes (Oguike et al., 2011). The findings of this study suggest that PocDBP-RII is an essential ligand for *P. ovale curtisi* blood-stage invasion. However, our results do not support the hypothesis that the same ligand is used by two *P. ovale* subspecies during invasion, as *P. ovale wallikeri* DBP is a pseudogene (Ansari et al., 2016; Rutledge et al., 2017). Whether the two *P. ovale* subspecies utilize the same ligands for invasion needs further exploration.

Due to the much lower morbidity, limited geographical distribution, and difficulty in the diagnosis of *P. ovale* by microscopic examination, the actual burden of *P. ovale curtisi* and *P. ovale wallikeri* is largely overshadowed by non-falciparum malaria parasites (Fuehrer et al., 2011; Bauffe et al., 2012; Fuehrer et al., 2012; Fuehrer and Noedl, 2014; Akerele et al., 2017; Joste et al., 2018). Improvements in molecular detection and surveillance studies have shown increasing evidence of *P. ovale* infection globally (Roucher et al., 2014; Trape et al., 2014; Li et al., 2016; Hawadak et al., 2021). However, along with surveillance studies, the invasion mechanism of these parasites also needs to be studied. The current study is the first experimental documentation of the function of one of the invasion ligands, PocDBP-RII, as determined by an *in vitro* study without *P. ovale curtisi* in *in vitro* culture system (Schuster, 2002). Other homologous invasion ligands of *P. ovale curtisi* compared to *P. falciparum* and *P. vivax* similarly need to be further comprehensively studied to elucidate the basic invasion mechanism of this parasite.

The most promising function of PvDBP is its reticulocyte binding and selection activity (Tran et al., 2005). In the current study, the high-likelihood binding functional domain from PocDBP was selected for the FACS-based binding assay with enriched reticulocytes. The PocDBP RII domain exhibited specific binding activity with reticulocytes rather than normocytes. This strong binding activity indicated that the PocDBP ligand might bind with an abundantly expressed reticulocyte receptor. One of the shortcomings in the current study is that anti-DARC antibody inhibition activity against the PocDBP-RII was not explored. Perhaps, which particular receptor is being used by the PocDBP-RII requires further exploration. PvDBP-RII showed stronger binding activity with reticulocytes than normocytes and bound specifically with DARCs, which are abundant in reticulocytes (Ovchynnikova et al., 2017). Similar to the PvDBP-RII protein, PocDBP-RII also showed neuraminidase resistance, which suggests that PocDBP-RII interacts with a non-sialic acid receptor on reticulocytes. Chymotrypsin-treated reticulocyte binding assays showed approximately 50% binding inhibition of PocDBP-RII compared with untreated control samples, which is consistent with the PvDBP-RII binding pattern (Han et al., 2016).

The 3-D structural prediction of PocDBP-RII shows a structure similar to that of PvDBP-RII. Although the sequence identity between PvDBP-RII and PocDBP-RII was low, the structural similarity indicated that PocDBP-RII might play a role in the invasion process. The reticulocyte preference of PocDBP-RII strengthens this hypothesis. Additionally, these findings could be indicative of cross-reactivity between PvDBP-RII and PocDBP-RII, although this requires experimental verification.

In summary, we found conserved domain sequences in the *pocdbp* gene in inter-*Plasmodium* spp. comparisons and observed specific binding to reticulocytes. This interaction inhibited by an immune IgG antibody and the binding specificity following enzyme treatment demonstrates the chymotrypsin sensitivity of PocDBP-RII. This finding suggests that PocDBP may be an essential ligand for reticulocyte invasion by *P. ovale curtisi.* Further similar studies with other blood-stage proteins of *P. ovale curtisi* may provide a clear picture of the complete invasion process of this neglected malaria parasite.

## Data Availability Statement

The original contributions presented in the study are included in the article. Further inquiries can be directed to the corresponding author.

## Author Contributions

MRH designed the study and wrote the paper. E-TH and SN supervised the study process. MRH, MHN, J-HH, and FM performed the experiments and analyzed the data. MHN, J-HH, FM, S-KL, J-HP, FL, WSP, and SN assisted in editing the manuscript. All authors contributed to the article and approved the submitted version.

## Funding

This study was supported by a National Research Foundation of Korea (NRF) grant funded by the Korean government (MSIP) (NRF-2021R1A2C2008235) and by the Basic Science Research Program through the National Research Foundation of Korea (NRF), funded by the Ministry of Science, ICT and Future Planning (NRF-2021R1A4A1031574).

## Conflict of Interest

The authors declare that the research was conducted in the absence of any commercial or financial relationships that could be construed as a potential conflict of interest.

## Publisher’s Note

All claims expressed in this article are solely those of the authors and do not necessarily represent those of their affiliated organizations, or those of the publisher, the editors and the reviewers. Any product that may be evaluated in this article, or claim that may be made by its manufacturer, is not guaranteed or endorsed by the publisher.
